# Wide-angle fluid reservoir thickness changes during short-term scleral lens wear

**DOI:** 10.1186/s40662-025-00443-3

**Published:** 2025-07-14

**Authors:** Feifu Wang, Stephen J. Vincent, Pauline Cho, Yi Shen, Zihao Sheng, Meixiao Shen, Jun Jiang

**Affiliations:** 1 National Clinical Research Center for Ocular Diseases, Eye Hospital, Wenzhou Medical University, Wenzhou, 325027 China; 2 Optometry and Vision Science, Centre for Vision and Eye Research, Queensland University of Technology, Brisbane, QLD Australia

**Keywords:** Fluid reservoir thickness, Scleral lens, Wide-angle OCT imaging, Customized software

## Abstract

**Background:**

To analyze the fluid reservoir thickness over the whole cornea during scleral lens settling using wide-angle optical coherence tomography (OCT) images and customized computer software.

**Methods:**

A total of 75 participants were recruited – 29 (myopes) with regular corneas and 46 with irregular corneas (35 with keratoconus, and 11 post-keratoplasty). All participants were fitted with customized scleral lenses and anterior segment OCT (Tomey Casia 2) images were taken 0, 30, 60, 120, and 240 min after lens application at the dispensing visit. Customized software was used to automatically segment the anterior cornea and the posterior surface of the scleral lens and determine the fluid reservoir thickness at 17 corneal regions across a 12 mm diameter.

**Results:**

Fluid reservoir thickness decreased over time (*P* < 0.001) following an exponential decay, with no differences observed over time between the three groups (*P* = 0.97). The reduction in fluid reservoir thickness over four hours varied slightly between the central (149 ± 9 μm), mid-peripheral (139 ± 11 μm), and peripheral regions (131 ± 15 μm), *P* = 0.046. The fluid reservoir was thinnest in the superior mid-periphery for both the myopia and post-keratoplasty groups, and centrally for the keratoconus group. The fluid reservoir was thickest inferiorly for all groups, with the greatest level of asymmetry observed along the vertical meridian.

**Conclusions:**

Fluid reservoir thickness decreased most rapidly during the first two hours of lens wear and followed an exponential decay for both regular and irregular corneas across all corneal locations. Fluid reservoir asymmetry was greatest along the vertical meridian with a thicker reservoir observed in the inferior corneal regions.

**Supplementary Information:**

The online version contains supplementary material available at 10.1186/s40662-025-00443-3.

## Background

The thickness and symmetry of the post lens fluid reservoir during scleral lens wear can influence both optical quality [1–3] and corneal physiology [4]. For example, a thicker central fluid reservoir has been associated with lens decentration [5, 6], induced prism [7], corneal oedema [8, 9], conjunctival prolapse [10, 11], and midday fogging [12]. On the other hand, insufficient fluid reservoir thickness can result in corneal complications [13, 14] arising from lens bearing. Therefore, an appropriate fluid reservoir thickness (central and peripheral) is a crucial aspect of scleral lens fitting to achieve the optimum comfort, vision, and physiological response.

The fluid reservoir thickness changes over time as the scleral lens gradually settles into the conjunctival tissue. The rate, or magnitude, of scleral lens settling is influenced by a number of factors, including the ocular condition, morphology of the cornea [15], conjunctiva, and sclera [16], scleral lens design [17, 18], fluid reservoir thickness [19, 20], and duration of lens wear (e.g. hours [15, 21–25] or months [26, 27]). Consequently, fluid reservoir thickness must be assessed after a period of lens wear to ensure corneal clearance during the initial fitting process and in the longer term.

Most research examining fluid reservoir dynamics has focused on the central corneal region [28]. The reduction in central fluid reservoir thickness appears to follow a two-phase exponential decay, with approximately 50% of lens settling occurring within the first 30 to 60 min of wear with stabilization after two hours [28]. While a limited number of studies have reported regional variations in both central and mid-peripheral fluid reservoir thicknesses during short-term scleral lens wear using Scheimpflug imaging [17] or optical coherence tomography (OCT) [20, 23, 29] up to a maximum distance of 4.5 mm from the corneal center, currently no studies have quantified changes in the fluid reservoir thickness across the entire cornea, beyond the mid-periphery.

Quantifying the fluid reservoir across the entire cornea is clinically important, as excessive thickness at the limbus can cause corneal hypoxia [30, 31] while insufficient clearance may lead to mechanical trauma [13]. Therefore, customized software [32] was used to automatically segment the fluid reservoir across the entire cornea (12 mm diameter) from OCT images obtained during scleral lens wear, in participants with regular and irregular corneas (keratoconus and post-keratoplasty).

## Methods

### Study design

This cross-sectional, non-randomized, controlled observational study was approved by the Ethics Committee of Wenzhou Medical University, and followed the Declaration of Helsinki. This study was approved by the Ethics Committee of Eye Hospital of Wenzhou Medical University (2021–109-K-93), and all participants provided informed consent.

### Participants

Eligible participants were recruited from two multicenter clinical trials conducted in accordance with Good Clinical Practice (GCP) guidelines in Wenzhou. Participants were fitted with customized scleral lenses (ICOMFiT Scleral Lens, Vision X, Medical Technology Co., Ltd., Shanghai, China) in one or both eyes, and had not worn scleral lenses previously. The final study cohort included 29 myopic participants (regular cornea group), 35 with keratoconus including 8 with moderate keratoconus and 27 with advanced keratoconus based on the Berliner classification of keratoconus (11 eyes had undergone corneal cross-linking), and 11 had undergone keratoplasty due to severe keratoconus (irregular cornea groups). Participants with regular corneas wore scleral lenses in both eyes, but only data from the left eye were analyzed. Participants with irregular corneas were fitted with scleral lenses in one or both eyes, depending on the clinical need (i.e., to one or both eyes as required). Only one eye was included in the analysis (the eye with the more severe condition if both eyes were fitted with lenses). The inclusion and exclusion criteria are summarized in Table 1.

**Table 1:** Inclusion and exclusion criteria

	Regular cornea group	Irregular cornea groups
Inclusion criteria	1. 18 to 40 years old	1. 18 to 40 years old
	2. Spherical equivalent refractive error within − 10.00 D to − 0.50 D	2. Keratoconus or keratoplasty
	3. Regular corneal astigmatism, astigmatism < 2.50 D	3. No diagnosis of glaucoma, cataract, retinal disease or infectious eye disease
	4. Best corrected visual acuity ≥ 1.0 (decimal visual acuity) in each eye	4. Eyes remained stable for more than six months after corneal cross-linking or remained stable for more than three months after post keratoplasty for those who had undergone suture removal
	5. Good ocular health with no contraindication to contact lens wear	
Exclusion criteria	1. Infectious eye disease 2. Systemic disease which can cause immunodeficiency or may affect rigid contact lens wear 3. Living or working environment unsuitable for wearing rigid lenses, windy or sandy environment, and environments with dispersed substances like, dust, chemicals, aerosols 4. History of contact lens allergy or allergy to contact lens care products 5. Pregnant or lactating women	

### Baseline clinical assessment

Non-cycloplegic subjective refraction was performed following the principle of maximum plus for maximum visual acuity to obtain the sphero-cylindrical refractive error and best-corrected visual acuity (BCVA). Scheimpflug imaging (Pentacam, OCULUS Optikgeräte GmbH, Germany**)** was used to measure the steep keratometry (K), flat K, and anterior chamber depth (ACD).

### Scleral lens fitting

Participants were fitted with diagnostic scleral lenses (Table 2) according to the manufacturer's fitting instructions. The lens diameter was determined based on the white-to-white (W-W) distance + 3.0 mm as a reference value. The initial sagittal height of the lens was selected using corneal sagittal height measurements obtained from OCT imaging as a baseline reference based on the manufacturer’s fitting guidelines, followed by iterative adjustments based on clinical fit assessment. The final sagittal height difference in the landing zone was determined by combining the Pentacam elevation map and the slit-lamp evaluation of the diagnostic lenses on the eyes after settling.

**Table 2:** Parameters of diagnostic scleral lenses

Parameters	Value
Brand	ICOMFiT (Visionxlab, Shanghai, China)
Lens design	Four-zone with toric landing zone
Material	Hexafocon A
Lens diameter	15.6 or 16.3 mm
Sagittal height	3800 to 4800 μm in 200 μm intervals
Back optic zone radius	6.90 to 8.45 mm
Center thickness	300 μm
Final lens diameter	72 participants wore lenses with a 15.6 mm diameter, 3 participants with a 16.3 mm diameter (one myopia, two keratoconus)
Final lens sagittal height Median (25th, 75th percentile)	Myopia group: 3800 (3800, 4000) μm Keratoconus group: 4000 (4000, 4200) μm Post-keratoplasty group: 4200 (4000, 4400) μm

The optimal back vertex lens power was determined through subjective over refraction after two hours of lens settling, and a customized lens with a toric landing zone was ordered for each eye to achieve an initial central fluid reservoir thickness between 200 to 400 μm, with landing zone alignment as observed by slit-lamp biomicroscopy.

In addition, assessments were performed with sodium fluorescein: (1) with the scleral lens in situ, fluorescein was used to evaluate whether there was any leakage of tears from the ocular surface into the lens at the landing zone; (2) after lens removal, to assess corneal and conjunctival staining (if present).

On the day the customized scleral lenses were dispensed, sequential OCT images were captured over four hours of lens wear. All OCT scans for each participant were performed by the same examiner. All scleral lenses were fitted by trained and experienced clinicians.

### OCT imaging and analysis

Ultra-wide angle (16 mm wide) anterior segment swept source OCT images (Tomey Casia 2, Tomey Corporation, Nagoya, Japan) centered on the corneal apex were captured at five different time points: shortly after lens application with sterile saline, then after 30, 60, 120, and 240 min of lens wear.

Both the upper and lower eyelids were gently retracted using a cotton swab or by instructing patients to look in specific directions to expose the central and relevant peripheral areas for scanning. Care was taken to minimize pressure on the globe and lens to avoid influencing the fluid reservoir thickness or lens position. Examiners were specifically trained in this procedure to ensure consistency across all participants.

The anterior segment 3D radial scan mode was used to collect 64 image frames within 1.8 s at each time point, of which 32 images were used for image recognition and 3D reconstruction using the customized software [32]. All OCT images were uniformly resized to a 1:1 aspect ratio with a resolution of 512 × 512 pixels for deep learning model training. After segmentation, the results were mapped back to the original image dimensions to preserve spatial accuracy. The brightness, contrast, and gamma values were not altered from the default settings of the OCT device.

**Fig. 1:**
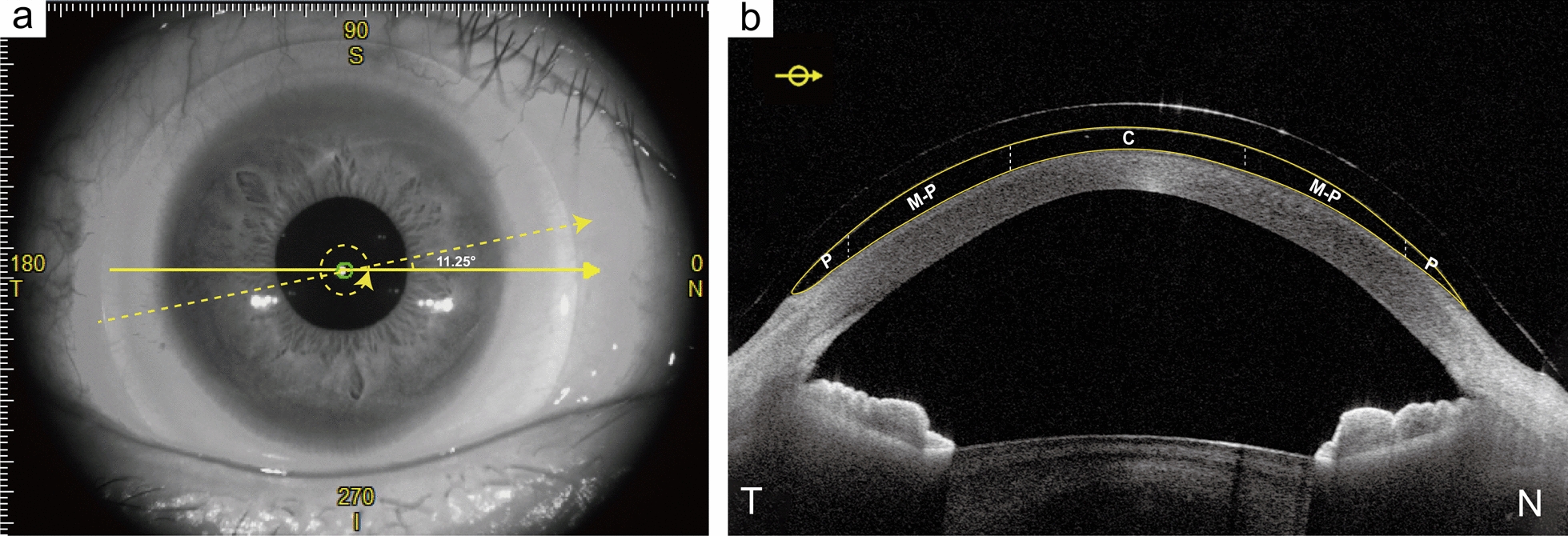
SS-OCT imaging and analysis of fluid reservoir boundaries. **a** SS-OCT images were taken with an image width of 16 mm using the anterior segment 3D radial scan mode. **b** Each OCT image was analyzed to detect the anterior and posterior boundaries of the fluid reservoir. The fluid reservoir was analyzed across central (C, 4 mm diameter), mid-peripheral (M-P, 3 mm annulus), and peripheral (P, 1 mm annulus) regions. OCT, optical coherence tomography; SS-OCT, swept-source optical coherence tomography. N, nasal; T, temporal; S, superior; I, inferior

**Fig. 2:**
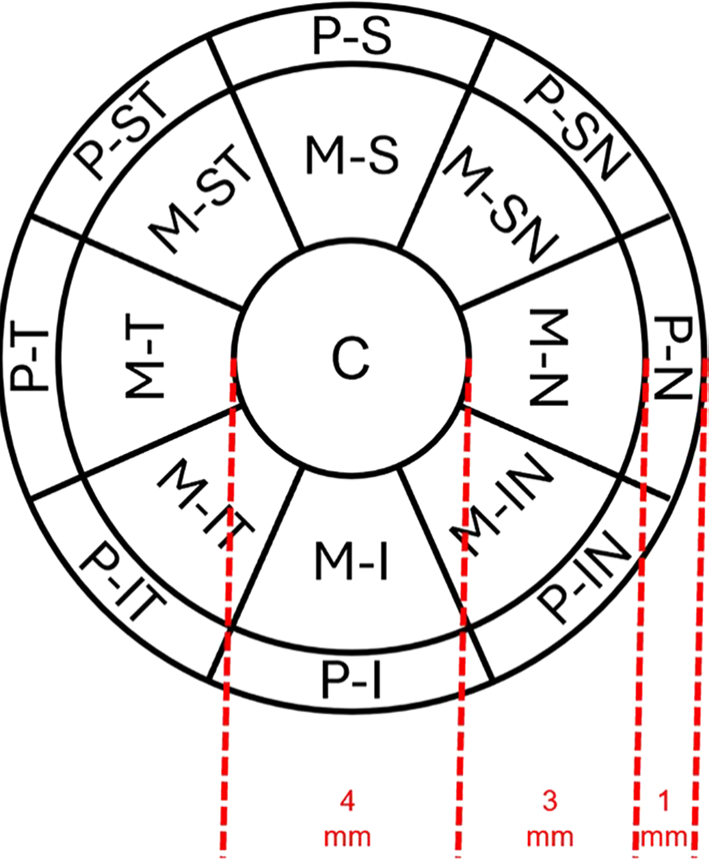
The reconstructed fluid reservoir was divided into three concentric circles with radii of 2 mm (central, C), 5 mm (3 mm mid-peripheral annulus), and 6 mm (1 mm peripheral annulus). The mid-peripheral (M) and peripheral (P) regions were further divided into 8 sections. N, nasal; T, temporal; S, superior; I, inferior; SN, superior nasa; ST, superior temporal; IN, inferior nasa; IT, inferior temporal

The image processing method used has been described previously [32, 33]. This customized (ScLNet) software has a high degree of precision (0.9678) and specificity (0.9965) compared to the manual delineation of OCT images [32]. In brief, each OCT image was analyzed to detect the back surface of the scleral lens and the anterior corneal epithelium (i.e., the anterior and posterior boundaries of the fluid reservoir; see Fig. 1). In this study, customized software was used to automatically interpret the processed images and quantify the fluid reservoir across the entire corneal region. Corrections were applied for refractive index, image depth and width in the calculation fluid reservoir thickness. The reconstructed fluid reservoir was analyzed across the central (4 mm diameter), mid-peripheral (3 mm annulus), and peripheral (1 mm annulus) regions, with the mid-peripheral and peripheral regions further segmented into eight sections as illustrated in Fig. 2.

### Statistical analysis

Statistical analyses were conducted using SPSS v.23.0. Data normality was assessed with the Shapiro–Wilk test. Baseline characteristics among the three groups were compared with one-way ANOVA. Fluid reservoir thickness was analyzed using repeated-measures ANOVA with time, region, and group as factors, applying the Greenhouse–Geisser correction if the assumption of sphericity was violated. Bonferroni correction was used for post hoc pairwise comparisons. Statistical significance was set at *P* < 0.05.

## Results

### Participants

The participant demographics and characteristics are summarized in Table 3.

**Table 3:** Characteristics of participants (mean ± SD, except for sex)

Parameter	Myopia (n = 29)	Keratoconus (n = 35)	Post-keratoplasty (n = 11)	*P* value
Age (years)	26.7 ± 3.8	28.6 ± 6.7	31.1 ± 4.2	0.02
Sex (F:M)	25:4	12:23	3:8	–
BCVA (logMAR)	− 0.10 ± 0.05	0.39 ± 0.15	0.41 ± 0.10	< 0.001
Sphere (D)	− 5.41 ± 3.06	− 6.97 ± 5.26	− 1.98 ± 4.19	< 0.001
Cylinder (D)	− 0.82 ± 0.54	− 3.48 ± 1.88	− 5.00 ± 2.93	< 0.001
SER (D)	− 5.53 ± 2.83	− 8.61 ± 5.21	− 4.48 ± 4.67	< 0.001
Flat K (D)	42.94 ± 1.32	49.24 ± 3.82	41.28 ± 2.43	< 0.001
Steep K (D)	44.05 ± 1.34	52.73 ± 3.82	46.40 ± 3.60	< 0.001
ACD (mm)	3.08 ± 0.20	3.36 ± 0.28	3.33 ± 0.41	0.001

*BCVA* = best-corrected visual acuity with spectacles; *SER* = spherical equivalent refraction; *flat K* = lattest keratometry reading; *steep K* = steepest keratometry reading; *ACD* = anterior chamber depth

### Change in fluid reservoir thickness

**Fig. 3:**
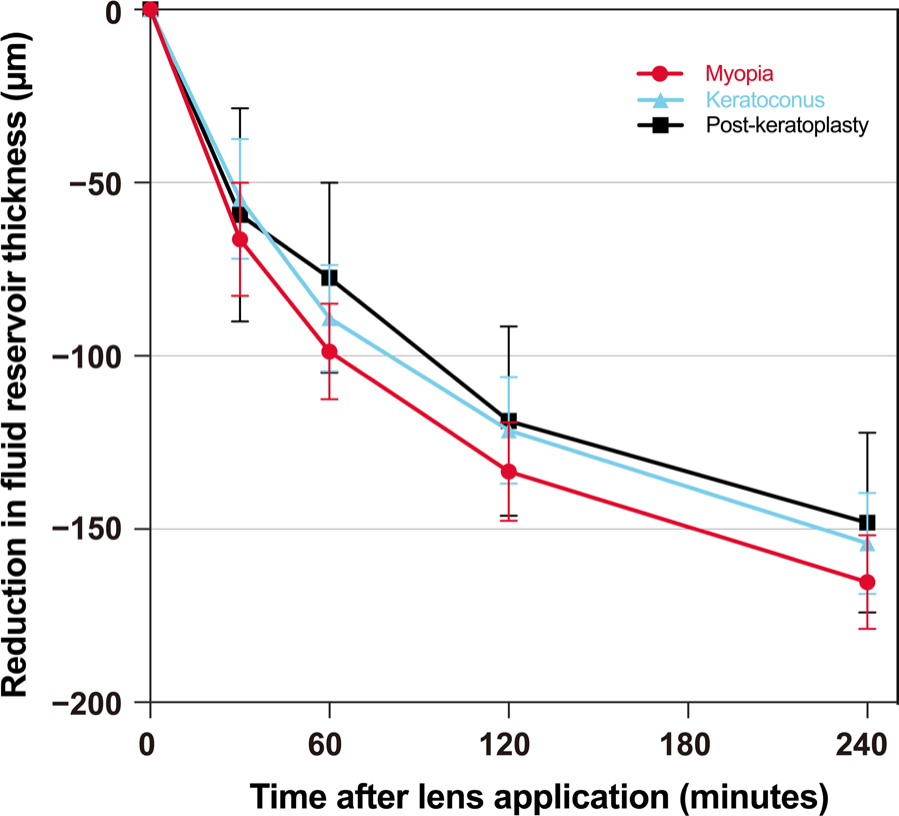
The mean reduction in fluid reservoir thickness (averaged across all regions) in each group (myopia, keratoconus, and post-keratoplasty) over four hours of scleral lens wear. Error bars represent standard error

The fluid reservoir thickness decreased significantly over four hours of scleral lens wear (*P* < 0.001) and was consistent across all regions in each group (time × group interaction, *P* = 0.97). Averaged across all regions, the fluid reservoir thicknesses decreased by 165 ± 14 μm (47% ± 3% reduction) in the myopia group, 154 ± 19 μm (32% ± 4% reduction) in the keratoconus group, and 148 ± 17 μm (32% ± 2% reduction) in the post-keratoplasty group over four hours (Fig. 3).

**Fig. 4:**
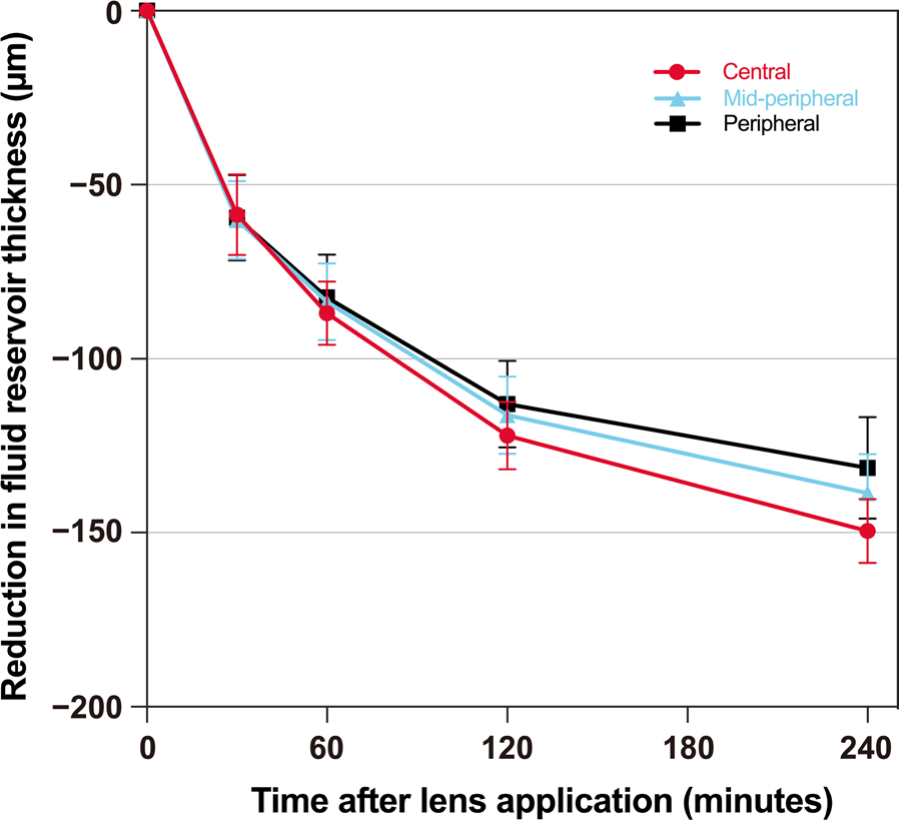
The mean reduction of post lens fluid reservoir thickness in the central, mid-peripheral, and peripheral regions averaged across all groups (myopia, keratoconus, and post-keratoplasty) over four hours of scleral lens wear. Error bars represent the standard error

The change in fluid reservoir thickness over time varied slightly between the central zone, mid-peripheral, and peripheral zones (*P* = 0.046, Fig. 4). Over four hours, the mean reduction in fluid reservoir thickness was 149 ± 9 μm for the central region, 139 ± 11 μm for the mid-peripheral region, and 131 ± 15 μm for the peripheral region. The greatest reduction in fluid reservoir thickness occurred within the first two hours of lens wear, accounting for approximately 73% of the total change over four hours across all regions.

**Fig. 5:**
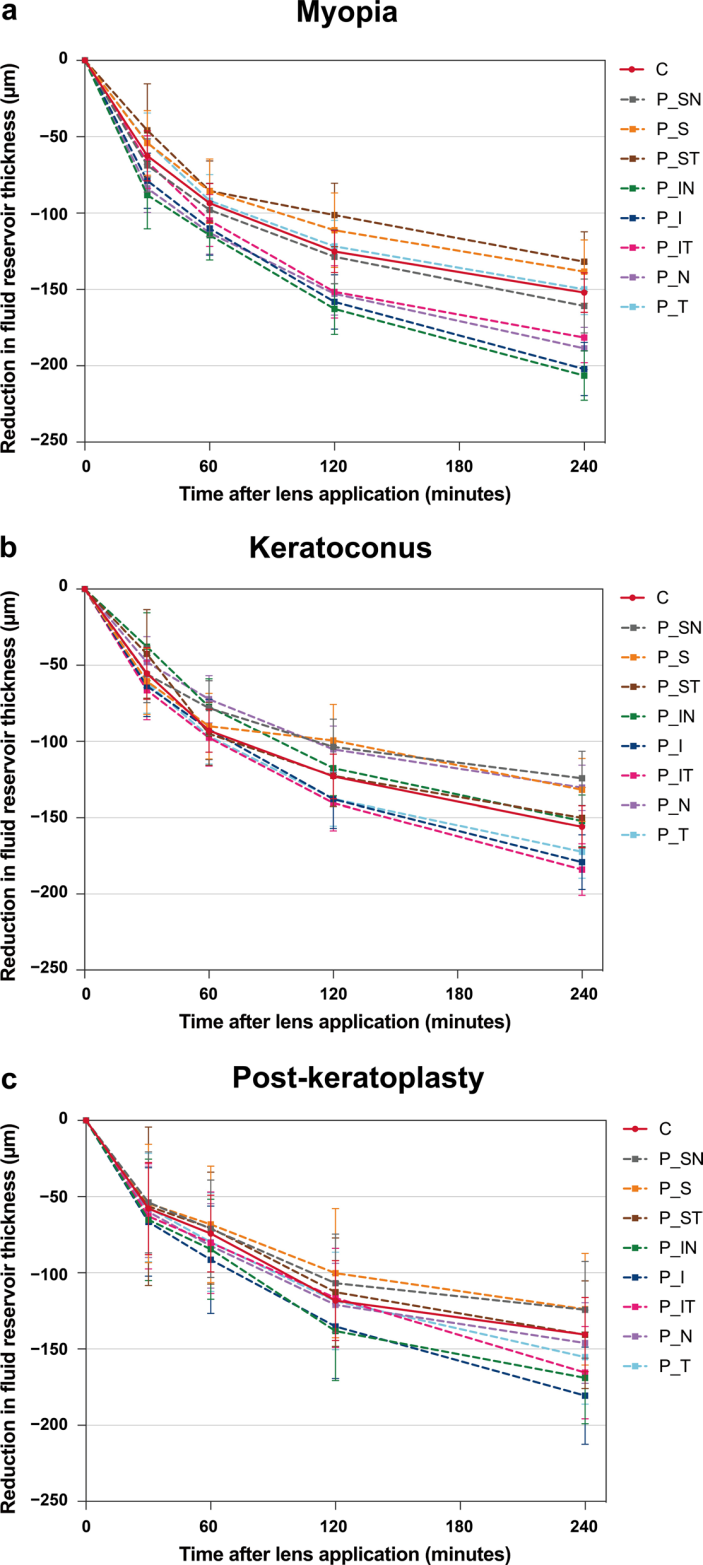
Reduction in post lens fluid reservoir thicknesses across the central and peripheral corneal regions in each group (myopia, keratoconus, and post-keratoplasty) over four hours of scleral lens wear. Error bars represent the standard error. C, central; P, peripheral; N, nasal; T, temporal; S, superior; I, inferior; SN , superior nasal; ST, superior temporal; IN, inferior nasal; IT, inferior temporal

Significant differences in fluid reservoir thickness were also observed across the different corneal regions within each group over four hours of lens wear (all *P* < 0.001, Fig. 5). At each time point, the thinnest fluid reservoir thickness in the myopia and post-keratoplasty groups was located in the mid-peripheral superior cornea (M-S, decreased from a mean of 274 ± 16 μm at baseline to 136 ± 13 μm in the myopia group, and from 369 ± 25 μm to 242 ± 20 μm in the post-keratoplasty group). In the keratoconus group, the fluid reservoir was consistently thinnest centrally, and on average decreased from 384 ± 12 μm at baseline to 228 ± 10 μm after four hours. For all three groups, the fluid reservoir was thickest in the inferior peripheral region (with baseline averages of 500 ± 19 μm, 563 ± 16 μm, and 639 ± 28 μm decreasing to 298 ± 19 μm, 384 ± 15 μm, and 458 ± 27 μm in the myopia, keratoconus, and post-keratoplasty groups, respectively; Fig. 5, Additional File 1 Figures S1).

## Discussion

This study utilized a novel, customized software based on a deep learning approach [32, 33] to automatically segment the post scleral lens fluid reservoir in wide-angle anterior segment OCT images across a 12 mm diameter. Consistent with previous studies [15, 18, 19, 23] examining the central corneal region, we found that the fluid reservoir decreased in a biphasic manner, with the majority of lens settling occurring within the first two hours and gradually plateauing thereafter. Interestingly, these dynamic changes were consistent across both regular and irregular cornea groups. Rathi et al. [15] also reported comparable settling after four hours of diagnostic scleral lens wear in patients with ocular surface disease (14% reduction) and corneal ectasia (17% reduction), compared with fluid thickness after one hour of lens wearing (19% to 26% in our study). Tran et al. [29] observed similar settling over four weeks in eyes with ocular surface disease and corneal ectasia but almost double the amount in eyes with corneal scarring. However, the duration of lens wear on the follow-up day was not controlled and the baseline fluid reservoir thickness was not reported.

Previous studies [15, 18–20, 23] examining fluid reservoir dynamics during scleral lens wear have been restricted to central or mid-peripheral measurements primarily due to imaging limitations, such as the instrument scan size or data availability (due to obscuration by the upper eyelid). The use of wide-angle imaging with eyelid retraction allowed simultaneous OCT imaging of the central and peripheral cornea, which revealed small differences in fluid reservoir thickness changes between the central (~ 40% reduction from the baseline reservoir thickness), mid-peripheral (~ 38%), and peripheral regions (~ 35%) over four hours (*P* = 0.046).

Changes in the fluid reservoir thickness are affected by several factors, including lens design [18], initial fluid reservoir thickness [19], and lens diameter [15, 19]. Kauffman et al. [18] demonstrated that different lens designs can lead to varying amounts of settling. Esen et al. [19] further showed that a lower initial fluid reservoir thickness and larger-diameter lenses result in less settling compared to smaller-diameter lenses or a thicker fluid reservoir. The greater fluid reservoir thickness and smaller lens diameter compared to other studies may explain the greater magnitude of lens settling observed in this study. However, when considered as a percentage reduction in fluid reservoir thickness, the results of the current study are broadly similar with previous work.

In the current study, the fluid reservoir was thinnest in the superior mid-peripheral region for the myopia and post-keratoplasty groups, and centrally for the keratoconus group, while the fluid reservoir was thickest inferiorly for all groups. This is most likely due to variations in corneal morphology between the groups, and the influence of inferior scleral lens decentration or tilt. Additionally, the eyelids may play a role in lens position and settling; during blinking and normal wear, the upper and lower eyelids can exert pressure that tends to push the lens downward and toward the ocular surface, further contributing to decentration and regional variations in settling. Consequently, the greatest asymmetry in fluid reservoir thickness was observed along the vertical meridian. A previous study [19] examining regional variations in fluid reservoir thickness from the center to mid-periphery in regular corneas over eight hours of lens wear reported a greater asymmetry along the horizontal meridian and attributed this to differences in nasal-temporal scleral elevation. These differences between the studies may relate to different lens designs or the ethnicity of the participants. For bilateral scleral lens corrections, this vertical asymmetry within the fluid reservoir is not a significant optical issue, but for monocular corrections can result in vertical diplopia due to an induced prismatic effect [7]. Significant variations in fluid reservoir thickness may also potentially act as a localized barrier to corneal oxygen delivery. Although scleral lenses could improve visual acuity in patients with irregular cornea [34], previous studies found in post-keratoplasty eyes that greater edema was observed inferiorly towards the graft host junction [35] and may be associated with corneal aberrations [36].

The major methodological advantage of the current study was the wide-angle OCT imaging that allowed measurement of the fluid reservoir out to a 12 mm diameter, compared to previous work which extended to only 9 mm in the corneal mid-periphery [29]. A limitation of the current study is the small sample size, especially in the post-keratoplasty group (n = 11), and the use of a single scleral lens design. Therefore, the results may not be generalized to all scleral lenses, particularly those with different landing zone configurations which may alter the symmetry of the fluid reservoir. In addition, the lenses were only worn for four hours. Although changes in central fluid thickness typically stabilized after four hours of lens wear [18], a longer period of observation (e.g., months or years of lens wear) would provide more insights into lens settling dynamics.

## Conclusion

The analysis of wide-angle OCT images with customized software facilitated the measurement of the fluid reservoir thickness across the entire cornea during scleral lens wear. The greatest change in fluid reservoir thickness was observed during the first two hours of lens wear, which was consistent across different groups and regions. In clinical practice, when assessing fluid reservoir thickness scleral lens practitioners must pay attention not only to central cornea, but also consider the vertical asymmetry of fluid reservoir. The observed changes in fluid reservoir thickness for optimally fitted scleral lenses were consistent for both regular and irregular corneas.

## Supplementary Information

Supplementary Material 1.

## Data Availability

The data presented in this study are available on request from the corresponding author with permission of Vision X, Medical Technology Co., Ltd., Shanghai, China.
