# Supplementary material for: Wide-angle fluid reservoir thickness changes during short-term scleral lens wear
Source: Eye Vis (Lond). 2025 Jul 14;12:27. doi: 10.1186/s40662-025-00443-3 (PMC12257854; doi:10.1186/s40662-025-00443-3)
Supplement: Supplementary file 1 — Supplementary Material 1. [file 40662_2025_443_MOESM1_ESM.docx]

**Additional File 1.**

**Figure S1.** Mean fluid reservoir thickness (μm, mean±SD) at each corneal region over the four-hour scleral lens wear for the myopia, keratoconus, and post-keratoplasty groups. N, nasal; T, temporal


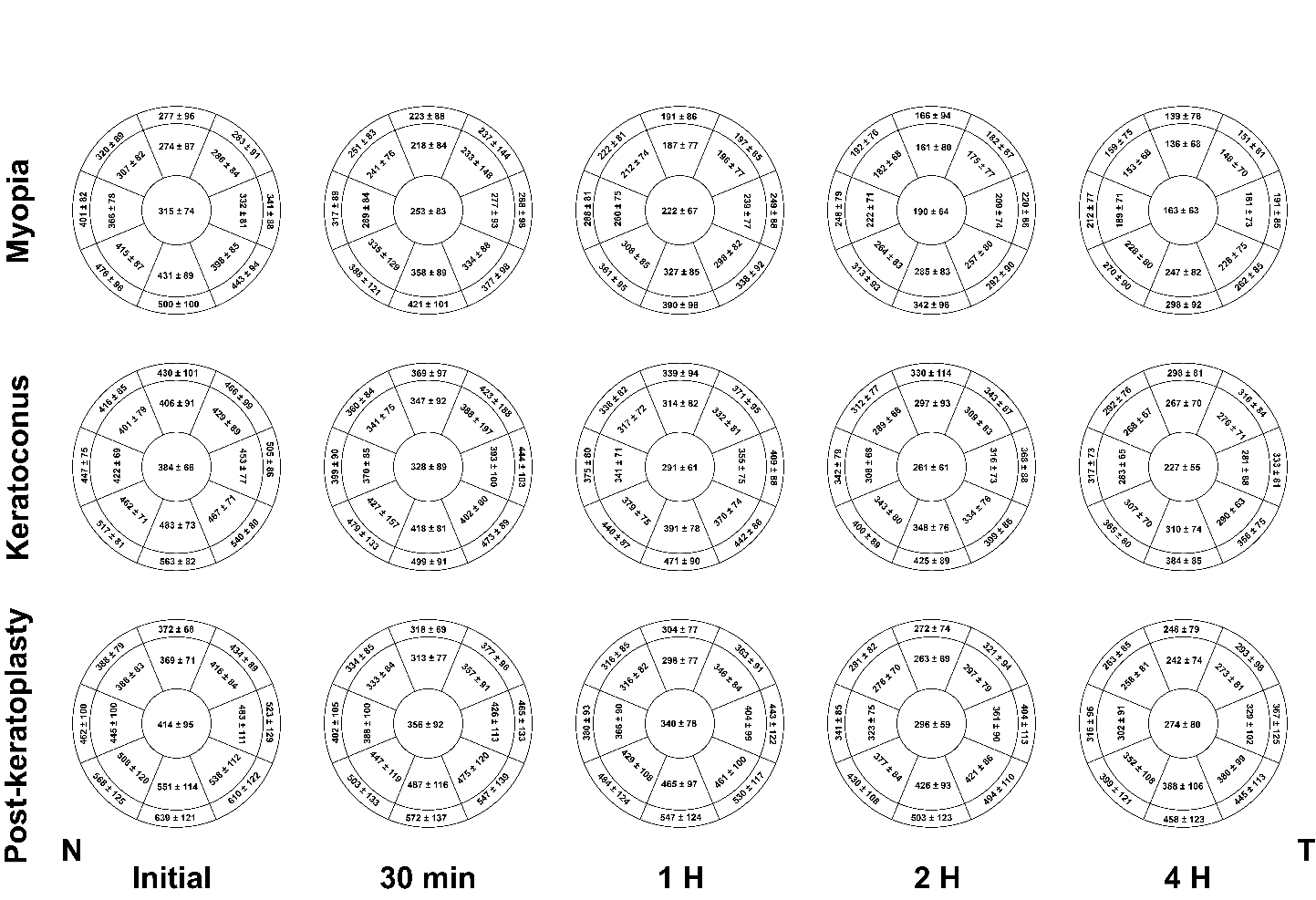


**Table S1.** Fluid reservoir thickness immediately after lens insertion and reduction in myopia group over four hours.

| **Parameter** | **Initial** | **30 min** | **1 H** | **2 H** | **4 H** |
| --- | --- | --- | --- | --- | --- |
| **(Mean ± SD)** |  |  |  |  |  |
| C | 315 ± 74 | 63 ± 89 | 93 ± 69 | 125 ± 74 | 152 ± 70 |
| M-SN | 307 ± 82 | 65 ± 86 | 94 ± 84 | 125 ± 85 | 153 ± 86 |
| M-S | 274 ± 87 | 56 ± 108 | 87 ± 100 | 114 ± 112 | 138 ± 98 |
| M-ST | 286 ± 84 | 53 ± 165 | 89 ± 94 | 111 ± 101 | 138 ± 94 |
| M-IN | 415 ± 87 | 80 ± 128 | 107 ± 76 | 150 ± 85 | 187 ± 77 |
| M-I | 431 ± 89 | 73 ± 86 | 104 ± 82 | 145 ± 84 | 183 ± 82 |
| M-IT | 398 ± 85 | 65 ± 85 | 100 ± 80 | 141 ± 83 | 171 ± 78 |
| M-N | 366 ± 78 | 78 ± 82 | 106 ± 71 | 144 ± 70 | 177 ± 71 |
| M-T | 332 ± 81 | 56 ± 101 | 93 ± 83 | 123 ± 82 | 151 ± 81 |
| P-SN | 320 ± 89 | 69 ± 94 | 98 ± 94 | 129 ± 95 | 161 ± 95 |
| P-S | 277 ± 96 | 54 ± 114 | 86 ± 114 | 111 ± 131 | 138 ± 112 |
| P-ST | 283 ± 91 | 46 ± 164 | 86 ± 106 | 101 ± 112 | 132 ± 106 |
| P-IN | 476 ± 96 | 88 ± 119 | 114 ± 88 | 163 ± 90 | 206 ± 87 |
| P-I | 500 ± 100 | 79 ± 97 | 110 ± 95 | 158 ± 96 | 202 ± 94 |
| P-IT | 443 ± 94 | 67 ± 93 | 105 ± 92 | 152 ± 92 | 181 ± 88 |
| P-N | 401 ± 82 | 84 ± 85 | 113 ± 76 | 153 ± 75 | 189 ± 74 |
| P-T | 341 ± 88 | 54 ± 103 | 92 ± 92 | 122 ± 92 | 150 ± 90 |

Initial: original fluid reservoir thickness immediately after lens insertion. 30 min/1 H/2 H/4 H: fluid reservoir thickness reductions compared to initial after 30 minutes/1 hour/2 hours/4 hours of lens wear. C = central; M = midperipheral; P = peripheral; N = nasal; T = temporal; S = superior; I = inferior; SN = superior nasal; ST = superior temporal; IN = inferior nasal; IT = inferior temporal.

**Table S2.** Fluid reservoir thickness immediately after lens insertion and reduction in the keratoconus group over four hours.

| **Parameter** | **Initial** | **30 mins** | **1 H** | **2 H** | **4 H** |
| --- | --- | --- | --- | --- | --- |
| **(Mean ± SD)** |  |  |  |  |  |
| C | 384 ± 74 | 55 ± 89 | 93 ± 69 | 123 ± 74 | 156 ± 70 |
| M-SN | 401 ± 82 | 60 ± 86 | 84 ± 84 | 112 ± 85 | 133 ± 86 |
| M-S | 406 ± 87 | 59 ± 108 | 91 ± 100 | 108 ± 112 | 138 ± 98 |
| M-ST | 429 ± 84 | 41 ± 165 | 97 ± 94 | 120 ± 101 | 153 ± 94 |
| M-IN | 462 ± 87 | 35 ± 128 | 83 ± 76 | 119 ± 85 | 155 ± 77 |
| M-I | 483 ± 89 | 65 ± 86 | 92 ± 82 | 135 ± 84 | 173 ± 82 |
| M-IT | 467 ± 85 | 66 ± 85 | 97 ± 80 | 133 ± 83 | 178 ± 78 |
| M-N | 422 ± 78 | 52 ± 82 | 80 ± 71 | 114 ± 70 | 139 ± 71 |
| M-T | 453 ± 81 | 60 ± 101 | 98 ± 83 | 137 ± 82 | 171 ± 81 |
| P-SN | 416 ± 89 | 56 ± 94 | 78 ± 94 | 104 ± 95 | 124 ± 95 |
| P-S | 429 ± 96 | 61 ± 114 | 90 ± 114 | 99 ± 131 | 132 ± 112 |
| P-ST | 466 ± 91 | 43 ± 164 | 95 ± 106 | 123 ± 112 | 150 ± 106 |
| P-IN | 517 ± 96 | 38 ± 119 | 77 ± 88 | 118 ± 90 | 152 ± 87 |
| P-I | 563 ± 100 | 64 ± 97 | 92 ± 95 | 138 ± 96 | 179 ± 94 |
| P-IT | 540 ± 94 | 67 ± 93 | 98 ± 92 | 140 ± 92 | 184 ± 88 |
| P-N | 447 ± 82 | 48 ± 85 | 72 ± 76 | 105 ± 75 | 130 ± 74 |
| P-T | 505 ± 88 | 62 ± 103 | 97 ± 92 | 138 ± 92 | 172 ± 90 |

Initial: original fluid reservoir thickness immediately after lens insertion. 30 min/1 H/2 H/4 H: fluid reservoir thickness reductions compared to initial after 30 minutes/1 hour/2 hours/4 hours of lens wear. C = central; M = midperipheral; P = peripheral; N = nasal; T = temporal; S = superior; I = inferior; SN = superior nasal; ST = superior temporal; IN = inferior nasal; IT = inferior temporal.

**Table S3.** Fluid reservoir thickness immediately after lens insertion and reduction in the post-keratoplasty group over four hours.

| **Parameter** | **Initial** | **30 min** | **1 H** | **2 H** | **4 H** |
| --- | --- | --- | --- | --- | --- |
| **(Mean ± SD)** |  |  |  |  |  |
| C | 414 ± 74 | 58 ± 89 | 74 ± 69 | 112 ± 85 | 141 ± 70 |
| M-SN | 388 ± 82 | 55 ± 86 | 72 ± 84 | 105 ± 112 | 130 ± 86 |
| M-S | 369 ± 87 | 56 ± 108 | 71 ± 100 | 118 ± 101 | 127 ± 98 |
| M-ST | 416 ± 84 | 58 ± 165 | 69 ± 94 | 131 ± 85 | 142 ± 94 |
| M-IN | 508 ± 87 | 61 ± 128 | 79 ± 76 | 126 ± 84 | 156 ± 77 |
| M-I | 551 ± 89 | 64 ± 86 | 87 ± 82 | 117 ± 83 | 163 ± 82 |
| M-IT | 538 ± 85 | 63 ± 85 | 78 ± 80 | 122 ± 70 | 159 ± 78 |
| M-N | 445 ± 78 | 57 ± 82 | 78 ± 71 | 122 ± 82 | 142 ± 71 |
| M-T | 483 ± 81 | 58 ± 101 | 79 ± 83 | 107 ± 95 | 154 ± 81 |
| P-SN | 388 ± 89 | 54 ± 94 | 71 ± 94 | 100 ± 131 | 124 ± 95 |
| P-S | 372 ± 96 | 54 ± 114 | 68 ± 114 | 113 ± 112 | 124 ± 112 |
| P-ST | 434 ± 91 | 56 ± 164 | 71 ± 106 | 138 ± 90 | 141 ± 106 |
| P-IN | 568 ± 96 | 65 ± 119 | 84 ± 88 | 135 ± 96 | 169 ± 87 |
| P-I | 638 ± 100 | 67 ± 97 | 91 ± 95 | 117 ± 92 | 181 ± 94 |
| P-IT | 610 ± 94 | 63 ± 93 | 80 ± 92 | 121 ± 75 | 165 ± 88 |
| P-N | 462 ± 82 | 60 ± 85 | 82 ± 76 | 118 ± 92 | 146 ± 74 |
| P-T | 523 ± 88 | 57 ± 103 | 80 ± 92 | 112 ± 85 | 155 ± 90 |

Initial: original fluid reservoir thickness immediately after lens insertion. 30 min/1 H/2 H/4 H: fluid reservoir thickness reductions compared to initial after 30 minutes/1 hour/2 hours/4 hours of lens wear. C = central; M = midperipheral; P = peripheral; N = nasal; T = temporal; S = superior; I = inferior; SN = superior nasal; ST = superior temporal; IN = inferior nasal; IT = inferior temporal.
